# Black Tea Increases Circulating Endothelial Progenitor Cells and Improves Flow Mediated Dilatation Counteracting Deleterious Effects from a Fat Load in Hypertensive Patients: A Randomized Controlled Study

**DOI:** 10.3390/nu8110727

**Published:** 2016-11-16

**Authors:** Davide Grassi, Richard Draijer, Casper Schalkwijk, Giovambattista Desideri, Anatolia D’Angeli, Sandro Francavilla, Theo Mulder, Claudio Ferri

**Affiliations:** 1 Department of Life, Health, and Environmental Sciences, University of L’Aquila, Viale S Salvatore, Delta 6 Medicina, 67100 L’Aquila, Italy; giovambattista.desideri@cc.univaq.it (G.D.); anatolia79@katamail.com (A.D.); sandro.francavilla@univaq.it (S.F.); claudio.ferri@cc.univaq.it (C.F.); 2 Unilever Research and Development, 3133 AT Vlaardingen, The Netherlands; richard.draijer@unilever.com (R.D.); Theo.Mulder@unilever.com (T.M.); 3 Department of Internal Medicine, CARIM School for Cardiovascular Diseases, Maastricht University Medical Center, 6229 HX Maastricht, The Netherlands; c.schalkwijk@maastrichtuniversity.nl

**Keywords:** black tea, flavonoids, endothelial function, circulating endothelial cells, hypertension

## Abstract

(1) Background: Endothelial dysfunction predicts cardiovascular events. Circulating angiogenic cells (CACs) maintain and repair the endothelium regulating its function. Tea flavonoids reduce cardiovascular risk. We investigated the effects of black tea on the number of CACs and on flow-mediated dilation (FMD) before and after an oral fat in hypertensives; (2) Methods: In a randomized, double-blind, controlled, cross-over study, 19 patients were assigned to black tea (150 mg polyphenols) or a placebo twice a day for eight days. Measurements were obtained in a fasted state and after consuming whipping cream, and FMD was measured at baseline and after consumption of the products; (3) Results: Compared with the placebo, black tea ingestion increased functionally active CACs (36 ± 22 vs. 56 ± 21 cells per high-power field; *p* = 0.006) and FMD (5.0% ± 0.3% vs. 6.6% ± 0.3%, *p* < 0.0001). Tea further increased FMD 1, 2, 3, and 4 h after consumption, with maximal response 2 h after intake (*p* < 0.0001). Fat challenge decreased FMD, while tea consumption counteracted FMD impairment (*p* < 0.0001); (4) Conclusions: We demonstrated the vascular protective properties of black tea by increasing the number of CACs and preventing endothelial dysfunction induced by acute oral fat load in hypertensive patients. Considering that tea is the most consumed beverage after water, our findings are of clinical relevance and interest.

## 1. Introduction

Hypertension is the leading risk factor for cardiovascular morbidity and mortality [1]. Several key mechanisms, including inflammation oxidative stress and endothelial dysfunction, play an important role in cardiovascular risk in late-life hypertension [1,2]. Over the past 25 years, a large body of clinical evidence has indicated a close relationship between the degree of endothelial dysfunction and clinical cardiovascular events in patients with cardiovascular risk factors, coronary heart disease, or both [3,4,5,6,7,8,9]. As a consequence, it has also been hypothesized that the reversal of endothelial dysfunction might slow down atherogenesis and improve individual cardiovascular prognosis [6,7,8,9].

Moreover, recent studies have shown that risk factors for vascular diseases are associated with a blunted capacity for repair of the endothelial damage evidenced by a dysfunction of bone marrow-derived circulating endothelial progenitor cells (EPCs) [10]. Indeed, a reduced ability of EPCs to proliferate ex vivo and to express an endothelial phenotype is associated with risk factors for coronary artery disease as well as endothelial dysfunction [10,11]. Cells grown under these conditions were formerly termed “early EPCs”, but are currently referred to as “circulating angiogenic cells” (CACs) [12,13]. CAC quantity and function are robust biomarkers of vascular risk for a multitude of diseases, particularly cardiovascular disease. Importantly, infused ex vivo-expanded CACs have shown a potential for improved endothelial function, either reducing the risk of events or enhancing recovery from ischemia [10,11,12,13,14].

Among various risk factors, hypertension is shown to be the strongest predictor of CAC migratory impairment [14]. Indeed, CACs serve as a cellular reservoir to replace dysfunctional endothelium and to form a cellular patch at the site of denuding injury [10,11,12,13,14]. 

Epidemiological studies have shown an inverse correlation between flavonoid-rich diets and cardiovascular disease [15,16]. Tea accounts for a major proportion of total flavonoid intake in a number of Western countries [15,16,17]. Increasing attention is currently being paid to the link between tea ingestion and a suggested lower incidence of cardiovascular events [16,17]. 

Some dietary intervention studies reported that both acute and chronic [18,19,20] black tea consumption increases NO-mediated flow-mediated dilation (FMD) in healthy volunteers as well as in patients with cardiovascular disease. In response to this, we performed a dose-finding study with black tea, showing that the daily consumption of even a single cup of tea (100 mg tea flavonoids) per day increased the FMD of healthy volunteers, and improving further with escalating dose [20].

In contrast, previous studies have shown that a meal rich in fat decreases FMD and negatively modifies vascular function [21,22]. However, the effects of tea on endothelial function under these challenging conditions has not been completely clarified. Indeed, Hodgson et al. [23], aiming to evaluate only the acute effects of tea compared with hot water in pharmacologically treated patients with coronary artery disease aged between 45 and 70 years, suggested that a mixed meal with tea was able to improve endothelium-dependent dilatation, but tea alone was not able to positively affect this parameter. We recently reported that black tea consumption lowered wave reflections and blood pressure in the fasting state and, during the challenging hemodynamic conditions after a fat load, in hypertensives [24]. The effects of high doses of green tea (1 L per day) on CACs have been reported for chronic heavy smokers [25], but could not be confirmed in patients with chronic renal failure [26]. However, the effects of black tea when consumed at a moderate dose on the number of CACs have not been examined. Therefore, the aim of the present study was to investigate the effects of black tea on endothelial function before and after an oral fat load and the number of functionally active CACs in a group of never-treated grade I essential hypertensives without additional cardiovascular risk factors.

## 2. Methods

### 2.1. Subjects

Nineteen never-treated hypertensive patients (7 males and 12 females; mean age ± standard deviation 51.5 ± 8.4 years) referring to our outpatient unit were recruited. Entry criteria were ≥18 and ≤75 years of age; systolic BP (SBP) between 140 and 159 mmHg or diastolic BP (DBP) between 90 to 99 mmHg; body mass index between 18 and 30 kg/m^2^. Individuals were excluded if they had an acute or chronic disease, including any type of metabolic abnormality, a major cardiovascular risk factor, or both. Patients on prescribed medication or dietary supplements within two weeks of entering the study as well as habitual smokers were excluded. Female participants not in a post-menopausal phase were also excluded. To further limit potential confounding factors, individuals were excluded if they reported daily intense sports activities (10 h/week), changes of 10% body weight within 6 months of entering the study, a current dietary treatment regimen, or participation in another clinical study within 3 months of entering this trial. The local Ethics Committee of L’Aquila approved the study on 20 December 2007 (ref: 53/2007), all clinical investigation have been conducted according to the principles expressed in the Declaration of Helsinki, and all participants gave written informed consent. Some of the study results have been previously reported [24].

### 2.2. Diagnosis of Arterial Hypertension 

Grade I essential hypertension was diagnosed according to the European Societies of Hypertension and Cardiology criteria [27]. For this purpose, before enrollment into the study, BP and heart rate were measured after 10 min in a seated position in a comfortable room. SBP/DBP for inclusion in the protocol were 140/90 and 160/100 mmHg on 4 visits performed at 1-week intervals. During each visit, BP was measured in quadruplicate with an oscillometric device (Omron 705 CP, Omron) at 2 min intervals. The first BP reading was discarded, and the average of the last 3 measurements recorded. On each occasion, BP was recorded by the same physician who was unaware of the study design, objectives, and results (i.e., was not a member of the research team). Secondary hypertension was excluded by clinical examination and appropriate tests.

### 2.3. Study Design

Participants were randomly assigned to consume a hot beverage containing 150 mg tea flavonoids or a placebo twice a day for eight days in a double-blind, cross-over design. During the two 8-day periods when the volunteers consumed the test products, they were asked to refrain from consuming tea, red wine, chocolate-based products, dietary supplements, and non-steroidal anti-inflammatory drugs (prostaglandin synthetase inhibitors). The wash-out period between the two treatments was 13 days. Vascular function was assessed on Day 7 of the intervention and was repeated on Day 8 with an oral fat load. Pre-weighted portions of the test products were supplied to the volunteers in coded sachets made from laminated aluminum foil. Compliance was checked by a questionnaire. Volunteers consumed two doses per day: approximately one hour before lunch and one hour before dinner. They were carefully instructed to add the contents of a sachet to 100–200 mL of boiling hot water and to stir the solution until the powder was completely dissolved. An addition of sugar, milk, lemon, etc. was not allowed. The product was consumed while it was still hot. On test days, the volunteers consumed the test product at the facility at the beginning of the test sequence (*t* = 0). 

The composition of the products is given in Table 1. Before the start of the study and after the first visit to the test facility, volunteers received the intervention products. The number of returned empty sachets was used to check compliance. On the morning of Day 7 and Day 8 of the two test periods, volunteers came to the facility early in the morning in a fasted state. On both days, baseline FMD measurements were performed. Volunteers were subsequently asked to consume their morning dose of the test product. On Day 7, all measurements were performed in a fasted state; on Day 8, volunteers consumed ultra-heat-treated whipping cream (1 g fat per kg bodyweight; for 100 mL of product: 27 g of fats, 12.6 g of carbohydrates, 2.1 g of proteins; energy value 302 kcal) approximately 30 min after consuming the test product. The range of energy intakes was between 649 and 951 kcal. On Day 7, FMD was performed before (*t* = 0) and 1, 2, 3, and 4 h after consumption of the test product in a supine position in a quiet, temperature-controlled (22 °C–24 °C) room by trained, certified staff who were blind to the study protocol. This procedure was repeated on Day 8, but with a fat load. The study (participant recruitment and follow-up) was conducted between 1 March 2008 and 1 March 2011. The study was stopped from April 2009 to May 2010 due to the earthquake that occurred in L’Aquila. The trial has been registered under number ISRCTN27687092 (http://www.isrctn.com/ISRCTN27687092).

### 2.4. Endothelial Function 

FMD of the brachial artery was measured before (baseline, *t* = 0) and 1, 2, 3, and 4 h after the tea intake during each scheduled visit. FMD of the brachial artery was assessed after 15 min at rest. FMD was always determined by the same physician, who was blinded to the study design and objectives. FMD of the brachial artery of the dominant arm was measured by ultrasonography (General Electric). The transducer was held at the same point throughout the scan by a stereotactic clamp. The arterial diameter was measured at approximately 5–10 cm above the elbow. After a 1 min baseline measurement, a cuff placed at the forearm below the elbow was inflated at 300 mmHg for 5 min and then released, resulting in a brief period of reactive hyperemia. The maximal dilation of the brachial artery was measured. The brachial artery diameter changes in response to increased blood flow were assessed for a further 3 min after cuff deflation. Using the FMDStudio system (QUIPU, Pisa, Italy) [28,29,30,31], the approximate position of the edges of the vessel was manually located before starting the examination. After this procedure, an automatic mathematical contour tracking operator locates and tracks the edges, supplying information about quality and the time course of measurements in real time [28,29,30,31]. Upon completion of the analysis, the device automatically generated a report with all recorded measurements. FMD is expressed as a percentage change from the baseline diameter. Endothelium-dependent vasodilation was considered the maximal dilation of the brachial artery induced by the increased flow [28,29,30,31]. 

### 2.5. Ex Vivo Expansion Assay and Characterization of CACs

Mononuclear cells (MNCs) were isolated using Ficoll density-gradient centrifugation from 20 mL of peripheral blood. MNCs washed three times in PBS and resuspended in an EBM-2 bullet kit (Cambrex Bio Science, Milano, Italy), supplemented with 20% fetal bovine serum (FBS) (Celbio, Milano, Italy), were seeded at 106 cells/cm^2^ on fibronectin-coated culture plates 24 wells (Becton Dickinson, Milano, Italy). After 4 days of culture at 37 °C and 5% CO_2_, the non-adherent cells were discarded by washing with phosphate buffer saline (PBS) (Celbio, Milano, Italy), while the adherent cells were maintained in culture for another three days and then underwent cytochemical analysis. Adherent cells were incubated with 1,1′-dioctadecyl-3,3,3′,3′-tetramethyl indocarbocyanine-labeled acetylated low-density lipoprotein (DiLDL) (Invitrogen, Milano, Italy) at a concentration of 2.4 μg/mL for 1 h at 37 °C. Cells were then fixed in 1% paraformaldehyde for 10 min and incubated with fluorescein isothiocyanate (FITC)-labeled Ulex europaeus agglutinin I (Sigma-Aldrich, Milano, Italy) at a concentration of 10 μg/mL for 1 h. Dual-staining cells positive for both DiLDL- and FITC-labelled UEA-1 were judged as functional CACs and were counted [13,32]. CACs were counted manually in 10 randomly selected microscopic fields by two independent investigators with an inverted fluorescence microscope (magnification ×20 times) (Zeiss, Oberkoken, Germany).

### 2.6. Haematochemistry and Blood Lipids

In all individuals, a routine hematochemical check was performed by standard methods after each active treatment phase. Fasting plasma glucose and insulin, serum total cholesterol, high-density lipoprotein (HDL) cholesterol, low-density lipoprotein (LDL) cholesterol, and triglyceride levels were assessed in the clinical chemistry laboratory using routine procedures. Plasma glucose and insulin values were used to calculate the index of insulin resistance, the homeostasis model assessment of insulin resistance (HOMA-IR) [24].

### 2.7. Biomarkers of Endothelial Dysfunction and Low-Grade Inflammation

Biomarkers of endothelial dysfunction (soluble vascular cell adhesion molecule 1 (sVCAM-1), soluble endothelial selectin (sE-selectin), and soluble intercellular adhesion molecule 1 (ICAM-1)) and of low-grade inflammation (C-reactive protein (CRP), serum amyloid A (SAA), interleukin (IL)-6, IL-8, IL1-β, tumor necrosis factor α (TNF-α), and sICAM-1) were measured via a multiarray detection system based on electrochemiluminescence technology (Meso Scale Discovery) in fasting EDTA plasma samples as previously described [33]. Endothelin-1 was measured by Elisa (R & D, R & D systems, Abingdon, UK).

### 2.8. Statistical Evaluation

#### Size of the Study Population

The power calculation was based on the change in FMD between the placebo and the active in the fasted state. Calculations were based on FMD data from a cross-over study in 60 slightly hypertensive subjects. FMD was measured at the end of each of the three interventions that each lasted for 4 weeks. The power calculation was based on an average FMD = 8.10%, within subject variance = 1.79% found in this study [20]. In the dose-finding study, a mean effect of 1.80% was detected at 400 mg/day of tea flavonoids, and the same effect was expected in the current study [20]. Based on these data, a group of 18 volunteers was required to detect a 1.8% difference between the placebo and the intervention (2-sided, alpha 0.05, and power 0.80). In order to account for a 10% drop-out, we included 20 volunteers. A randomization scheme was prepared by a statistician using computer generated random numbers. Randomization was done before the start of the study by assigning treatment orders to subject numbers with a block size of two and an allocation ratio of 1:1. Sachets with the test products were labeled with subject number and treatment period. Treatments were supplied in sealed in envelopes labeled with the subject number. These were opened in consecutive order by each participant. Participants, statisticians, care providers, and those assessing the outcome of the study were all blinded. The black tea and the placebo were as similar as possible in taste and appearance. 

Data analysis was performed using the SAS software (SAS Institute, Cary, NC, USA, version 9.1). Descriptive analysis consisting of distribution statistics (number of available observations, mean and standard deviations) were presented for continuous data. Differences between the experimental groups and the placebo of the acute effect after one week intervention were evaluated by means of an analysis of covariance, integrating the data of 1, 2, 3, and 4 h after test product ingestion. The statistical model included the covariables baseline (*t* = 0), gender, age, and BMI. Fixed factors included in the model include treatment, period, day, time, and all their interactions. Other interactions in the model include baseline–treatment, baseline–period, and baseline–time. The chronic effects, after one week intervention, were calculated from the baseline data using a similar model, without the baseline covariable.

The influence of the interventions was assessed within each individual. Therefore, the variation due to the differences between individuals was separated from the relevant error variance in the analysis. A Dunnett test was performed in order to correct for multiple testing between the active treatment and the placebo with and without a fat load. The statistical analysis results are presented as LSmeans ± standard error (SE) or median (interquartile range) for data with a skewed distribution (plasma biomarkers). Statistical tests are two-sided for all analyses with a significance level of 0.05.

## 3. Results

Baseline characteristics of the study participants are given in Table 2. 

Of the 20 individuals enrolled, 19 completed the study. One individual dropped out due to personal circumstances and was excluded from the statistical analysis. Compliance was 100% in all the volunteers for all the study phases.

### 3.1. Endothelial Function

**Figure 1:**
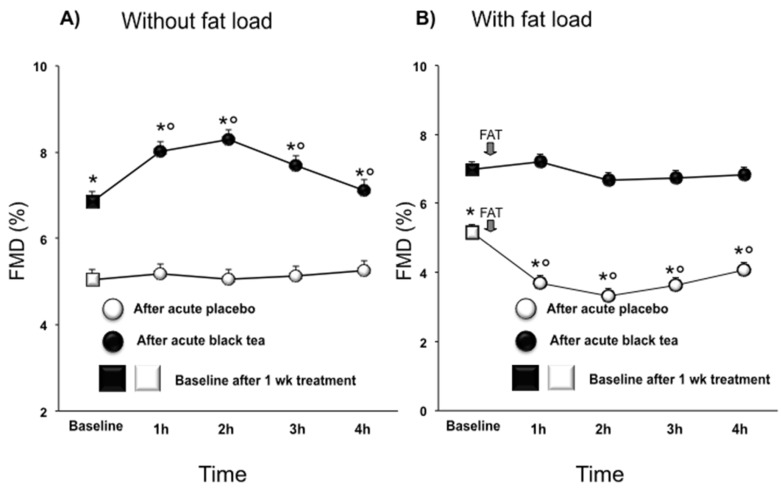
Effect of a 1 week placebo (**white**) and black tea (**black**) administration on baseline (square) values (**A**,**B**) of flow-mediated dilation (FMD) and acute effects without and with fat load (**A**,**B**). Data are presented as LSmeans ± SE. In all panels, vertical lines indicate SE, and asterisks (*) indicate significant differences with respect to the placebo phase (**A**,**B**), while circles (°) indicate significant differences from the baseline values (**A**,**B**). Differences are considered significant when *p *< 0.05.

Compared with the placebo, one week of black tea ingestion increased baseline FMD (Day 7: 5.0% ± 0.25% vs. 6.8% ± 0.25%; Day 8: 5.2% ± 0.24% vs. 7.0% ± 0.24%; *p* < 0.0001 for both (Figure 1A,B). On Day 7, acute black tea administration additionally increased FMD (from 6.8% to 7.5%, 7.8% and 7.2% *p* < 0.0001) at 1, 2, and 3 h after intake, respectively. No significant changes were observed after acute placebo administration. On Day 8, in the placebo group, FMD fell from 5.2% at baseline to 4.4%, 4.1%, 4.4%, and 4.8% at 1, 2, 3, and 4 h (*p* < 0.0001), respectively, after the fat load (Figure 1B). This drop in FMD was almost completely prevented by concomitant intake of tea (Figure 1B). 

### 3.2. Functional CACs

**Figure 2:**
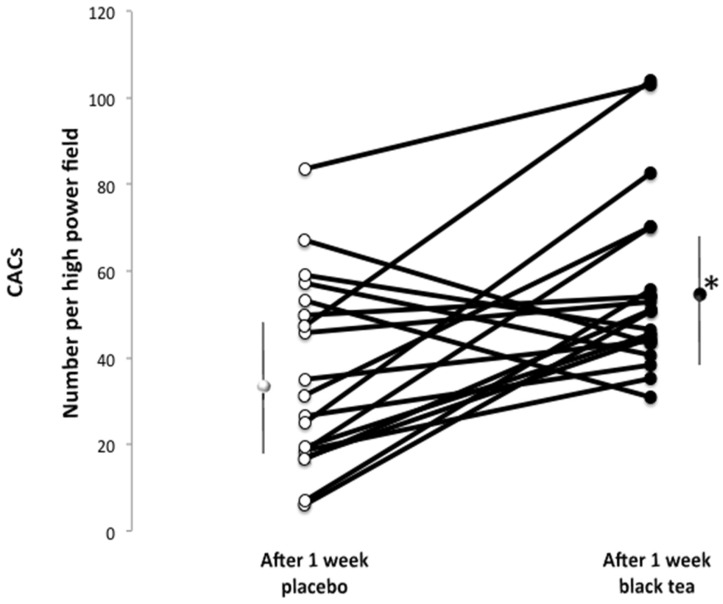
Absolute numbers of functional circulating angiogenic cells (CACs) per high-power field fluorescence microscopy (×20) before and after 1 week of treatment with placebo (**white**) and black tea (**black**) administration. Data are presented as LSmeans ± SE. Vertical lines indicate SD, and asterisks (*) indicate significant differences with respect to the placebo phase. Differences are considered significant when *p *< 0.05.

Compared with the placebo, the number of functionally active CACs indicated as double positive cells per high-power field was significantly higher after black tea intake (black tea mean: 56.0 ± 21; placebo mean: 36 ± 22 number of cells per field; *p* = 0.006) (Figure 2). 

### 3.3. Plasma Lipoproteins, Glucose, Insulin, and Biomarkers 

No significant differences were observed between the two treatments for plasma concentrations of biomarkers of endothelial dysfunction and low-grade inflammation (Table 3) and lipoproteins, glucose, and insulin (Table 4). 

## 4. Discussion

Our study showed that, in grade I hypertensive patients, one-week consumption of black tea resulted in a significant improvement in endothelium-dependent FMD, with maximal response two hours after acute intake. This effect was observed with a moderate dose, the equivalent of two cups of tea per day. Moreover, the consumption of tea counteracted a fat challenge-induced impairment of FMD. The protective properties of tea were also reflected by a significantly increased number of CACs, circulating cells capable of repairing the vessel wall. These results suggest that the consumption of tea may improve or even protect endothelial function under challenge conditions. 

Vascular endothelial dysfunction is determined by both genetic and environmental factors that cause decreased bioavailability of the vasodilator nitric oxide (NO). Under physiologic conditions, the endothelium regulates vascular tone via the balanced production of vasodilating substances such as NO. Impaired endothelium-dependent vasodilation has been reported after a fat load, probably by an increased production of oxygen-derived free radicals and a quenching of NO [22,34]. In particular, it has been observed [34] that acute fat load administered orally or intravenously significantly increased BP, impaired endothelial function, and activated the sympathetic nervous system via mechanisms not likely depending on changes in leptin, glucose, and insulin levels in obese healthy subjects. Thus, fat load has deleterious hemodynamic effects on obese subjects as well as on other conditions of cardiovascular risk, which may be involved in endothelial function [34,35]. Nevertheless, the effect of a high fat meal on endothelial function is not completely clear cut; indeed, Hodgson et al. [23] showed that a mixed meal with tea was able to improve endothelium-dependent dilatation, but tea alone was not able to positively affect this parameter. However, that study only evaluated the acute effects of tea compared with hot water, with a mixed meal (not specifically a fat load) in pharmacologically treated patients (with a number of putative confounding treatments: the use of aspirin, statins, or specific antihypertensive medications including angiotensin-converting enzyme inhibitors, angiotensin II receptor blockers, beta-blockers, calcium channel entry blockers, and diuretics) at very high cardiovascular risk (with coronary artery disease) aged between 45 and 70 years.

The increased FMD in fasted conditions and preserved postprandial FMD after tea intake may explain our observed findings on BP and arterial hemodynamics as described previously [23]. Taken together, our findings imply that the oral fat load may lead to a transient loss of NO bioavailability and transient vascular damage reflected in endothelial dysfunction and increased peripheral vascular tone and arterial stiffness. 

The severity of endothelial dysfunction correlates with the development of coronary artery disease and predicts future cardiovascular events [3,4,5,6,7,8]. Thus, endothelial dysfunction may be considered a strategic target in the treatment of hypertension. The flavonoids in tea may increase or preserve the bioavailability of NO by decreasing the formation or scavenging of reactive oxygen and nitrogen species, increasing NO synthase activity, or both [16]. Besides the functionality of the endothelium, the morphologic integrity of the monolayer constituted by resident vascular endothelial cells plays a pivotal role in the maintenance of a number of vascular functions [36]. Anatomical or functional interruption continuity of the endothelial barrier is considered a fundamental step during the atherogenetic process [36]. The integrity of the vascular endothelial barrier is continuously maintained by the rapid migration of resident endothelial cells toward wounded areas of the endothelium [35]. In addition to the migratory capability of resident vascular endothelial cells neighboring a damaged area, a growing body of evidence suggests that CACs also play a critical role in restoring the integrity of the endothelial monolayer [36]. Cardiovascular risk factors are associated with a reduced number of CACs [36], whereas, in turn, statins, angiotensin-converting enzyme inhibitors, and angiotensin II type 1 receptor blockers have been reported to increase the number of CACs [36]. Low levels of CACs in patients with increasing cardiovascular risk, such as hypertension, could have several mechanistic causes [10,11,14]. The most likely mechanism affected by tea flavonoids is the counteraction of oxidative stress, increasing NO bioavailability and FMD and decreasing BP levels in grade I hypertensive patients [24]. This hypothesis is supported by a study of Hill et al. [10] reporting a strong correlation between the number of CACs and the subjects’ combined Framingham risk factor score (*r* = −0.47). Further, measurement of FMD also revealed a significant relationship between endothelial function and the number of EPCs (*r* = 0.59, *p* < 0.001), where the level of CACs was a better predictor of vascular reactivity than the presence or absence of conventional cardiovascular risk factors [10]. In addition, CACs from subjects at high risk for cardiovascular events had higher rates of in vitro senescence than cells from subjects at low risk. A study by Bocchio et al. [30] showed that the number of CACs was significantly reduced in patients with cardiovascular risk compared with controls (*p* < 0.0001). The percentage variation of CACs and of FMD after treatment was significantly associated with the presence of endothelial dysfunction at baseline. The close relationship between FMD and the number of CACs may be explained by the important role of eNOS in the mobilization of progenitor cells from bone marrow [37].

A number of biomarkers were analyzed in this study in an attempt to provide more mechanistic insight; however, as blood lipids and markers of endothelial dysfunction and low-grade inflammation were not modified by the intervention, these results do not provide links to potential underlying mechanisms. 

The finding that tea improves FMD and increases the number of CACs has previously been reported by Kim et al. [25]. They observed in smokers that circulating and cultured angiogenic cells increased rapidly two weeks after green tea consumption. FMD correlated with CAC counts (*r* = 0.67) before and after treatment (*r* = 0.60). Of note, the flavonoid dose used by Kim et al. [24] was about three times higher than in the present study, and their study did not have a placebo group. Our hypothesis is that flavonoids in black tea increase the functionally active CACs numbers by the activation of eNOS, increasing NO bioavailability and concomitantly increasing FMD.

## 5. Conclusions

Therefore, the consumption of black tea in moderation improved FMD under fasted conditions and preserved FMD under postprandial conditions in hypertensive patients. The number of CACs doubled after the consumption of tea. Increased eNOS activity and NO bioavailability may be a common factor affected by tea flavonoids that explain both phenomena. Considering that tea is the most consumed beverage after water, our findings are of clinical relevance and interest, and our observations may have an impact on human health with important clinical consequences.

## Figures and Tables

**Table 1:** Composition of the test products (mg per dose).

	Placebo	Tea
Tea solids	0	497.5
Polyphenols	0	150
Catechins	0	12.1
Theaflavins	0	5.0
Gallic acid	0	4.5
Caffeine	37.3	37.3
Theanine	0	9.1
Caramel colour	90	0
Tea flavor	10	0
Sucrose	1363	1403
Total weight of sachet	1500	1900

**Table 2:** General characteristics of the study population (mean ± SD).

Characteristic	Value
Number of subjects (total/males)	19/5
Age (years)	51.3 ± 8.2
BMI (kg/m^2^)	27.1 ± 1.2
Body weight (kg)	73.7 ± 7.2
LDL-cholesterol (mg/dL)	141.1 ± 27.3
HDL-cholesterol (mg/dL)	45.7 ± 8.2
Triglycerides (mg/dL)	116.8 ± 38.1
Plasma glucose (mg/dL)	86.8 ± 7.3
Plasma insulin (μU/mL)	11.3 ± 5.4

**Table 3:** Plasma concentrations of biomarkers of endothelial dysfunction and low-grade inflammation.

	CRP (mg/L)	SAA (mg/L)	sICAM (ng/mL)	IL-1ß (ng/L)	IL-6 (ng/L)	IL-8 (ng/L)	TNF-α (ng/L)	sVCAM (ng/mL)	E-sel (ng/mL)	ET-1 (ng/L)
Placebo	1.6	1.6	211	1.5	2.3	12.5	9.8	320	158	1.4
	0.1–12.2	0.4–8.9	132–311	0.2–19.6	0.3–19.4	0.3–2788	4.7–25.5	163–598	35–480	0.8–2.3
Tea	0.9	1.6	201	1.7	2.5	8.9	10.8	337	125	1.5
	0.1–16.1	0.3–11.4	150–253	0.2–17.0	0.7–7.8	0.5–696	6.0–18.4	180–576	55–347	1.0–2.1

Values are presented as median with 1st and 3rd quartile due to skewed nature of the data (not normal distributed). EDTA-plasma samples were derived from fasted blood samples. Values are not significantly different (*p* > 0.1) between interventions. CRP: C-reactive protein; SAA: serum amyloid A; sICAM: soluble intercellular adhesion molecule 1; IL-1β: Interleukin-1β; IL-6: interleukin-6; IL-8: interleukin-8; TNF-α: tumor necrosis factor α; sVCAM: soluble vascular adhesion molecule 1; E-sel: E-selectin; ET-1: endothelin-1.

**Table 4:** Plasma concentrations of lipoproteins, glucose, insulin, and biomarkers.

	TC (mg/dL)	LDL (mg/dL)	HDL (mg/dL)	TG (mg/dL)	Glucose (mg/dL)	Insulin (mU/L)
Placebo	211.1 ± 4.3	144.9 ± 4.1	46.1 ± 2.0	112.7 ± 7.8	86.8 ± 1.4	11.9 ± 0.9
Tea	208.9 ± 4.3	138.5 ± 4.1	45.3 ± 2.0	119.5 ± 7.8	86.8 ± 1.4	10.5 ± 0.9

Values are in LSmeans ± SE. EDTA-plasma samples were derived from fasted blood samples. Values are not significantly different (*p* > 0.1) between interventions. TC: total cholesterol; LDL: low-density lipoprotein; HDL: high-density lipoprotein; TG: triglycerides.
